# Self-mapping the longitudinal field structure of a nonlinear plasma accelerator cavity

**DOI:** 10.1038/ncomms12483

**Published:** 2016-08-16

**Authors:** C. E. Clayton, E. Adli, J. Allen, W. An, C. I. Clarke, S. Corde, J. Frederico, S. Gessner, S. Z. Green, M. J. Hogan, C. Joshi, M. Litos, W. Lu, K. A. Marsh, W. B. Mori, N. Vafaei-Najafabadi, X. Xu, V. Yakimenko

**Affiliations:** 1 Department of Electrical Engineering, University of California Los Angeles, Los Angeles, California 90095, USA; 2 SLAC National Accelerator Laboratory, Menlo Park, California 94025, USA; 3 Department of Physics, University of Oslo, Oslo 0316, Norway; 4 Department of Physics and Astronomy, University of California Los Angeles, Los Angeles, California 90095, USA; 5 LOA, ENSTA ParisTech, CNRS, Ecole Polytechnique, Université Paris-Saclay, Palaiseau 91762, France; 6 Department of Engineering Physics, Tsinghua University, Beijing 100084, China

## Abstract

The preservation of emittance of the accelerating beam is the next challenge for plasma-based accelerators envisioned for future light sources and colliders. The field structure of a highly nonlinear plasma wake is potentially suitable for this purpose but has not been yet measured. Here we show that the longitudinal variation of the fields in a nonlinear plasma wakefield accelerator cavity produced by a relativistic electron bunch can be mapped using the bunch itself as a probe. We find that, for much of the cavity that is devoid of plasma electrons, the transverse force is constant longitudinally to within ±3% (r.m.s.). Moreover, comparison of experimental data and simulations has resulted in mapping of the longitudinal electric field of the unloaded wake up to 83 GV m^−1^ to a similar degree of accuracy. These results bode well for high-gradient, high-efficiency acceleration of electron bunches while preserving their emittance in such a cavity.

## Abstract (web summary)

Extraction of ultra-low emittance bunches is an issue to be addressed for future applications of plasma wakefield accelerators. Here, the authors show that the field structure of the plasma could be suitable for this, by measuring the field's longitudinal variation produced by a relativistic electron bunch.

## Graphical abstract

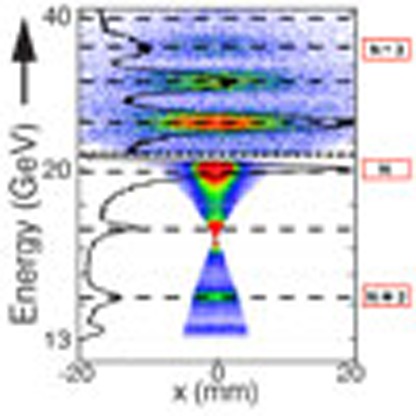

Adense, ultra-relativistic electron bunch propagating through an uniform plasma can produce a highly nonlinear wake that can be employed for accelerating a second, trailing bunch in a scheme known as the plasma wakefield accelerator (PWFA). Recent work has shown that a PWFA cavity (the wake) can accelerate a low-energy-spread electron bunch containing a significant charge at both high gradients and high-energy extraction efficiency^1^—necessary conditions for making future particle accelerators both compact and less expensive. In addition to this, the next important issues that must be addressed are the generation^2^,^3^, acceleration^4^ and extraction^5^ of ultra-low emittance bunches from plasma accelerators. Such high quality bunches are ultimately essential for obtaining extremely bright beams for future light sources^6^ and high luminosities for future particle colliders^7^.

Here we demonstrate that a highly nonlinear, three-dimensional (3D) PWFA cavity has the internal field structure needed to accelerate electrons with little emittance growth. We do this by analysing, on a single-shot basis, the observed transverse (*r*) oscillations of the longitudinal (*ξ*=*ct*−*z*) electron-bunch ‘slices' as they gain or lose energy and comparing these measurements with theory and particle simulations. We find that, for much of the PWFA cavity that is devoid of plasma electrons^8^, the transverse force *F*_*r*_=*e*(*E*_*r*_−*B*_*θ*_) is uniform with *ξ* to within ±3% root mean square (r.m.s.). Here, *E*_*r*_ is the radial electric field and *B*_*θ*_ is the azimuthal magnetic field. In this case, as a consequence of the well-known Panofsky–Wenzel (P–W) theorem in accelerator physics^9^,^10^,^11^, ∂_*ξ*_*F*_*r*_=∂_*r*_*F*_*z*_=0 to this degree of uncertainty. Furthermore, we have accurately mapped the longitudinal electric field *E*_*z*_(*ξ*) of the wake. Precise knowledge of this field/force structure is essential for determining the optimal shape and placement of the trailing bunch to load the wake so as to maximize the energy extraction efficiency^12^ while retaining the narrow energy spread with little emittance growth; a major challenge for plasma accelerators. We note that beam loading modifies the longitudinal field structure of wake and may lead to a growth of head-to-tail instabilities^13^,^14^.

Unlike the traditional metallic accelerating cavities powered by radio frequency waves, plasma wakefields are highly transient (∼few ps) and microscopic (∼100 μm) structures that propagate along with the drive pulse at the speed of light^15^. Spectral interferometry^16^ and shadowgraphy^17^,^18^ have previously been used to give information about the shape, wavelength, lifetime and radius of the nonlinear plasma wakes produced by intense laser pulses. However, no detailed information about the field structure within the primary accelerating cavity has been obtained in these studies.

The P–W theorem for plasma wakefields is just a statement of the relationship between ∂_*ξ*_*F*_*r*_ and ∂_*r*_*F*_*z*_. It can be readily derived (assuming stationary ions and under the quasi-static approximation) using the pseudo-potential Ψ=(*ϕ*–(*v*_*φ*_/*c*)*A*_*z*_), where *A*_*z*_ is the *z*-component of the vector potential and *ϕ* is the scalar potential. For a wake with phase velocity *v*_*φ*_=c, the longitudinal and transverse forces on the relativistic particle in the wake are given by *F*_*z*_=−*e*∂_*z*_Ψ and *F*_⊥_=−*e*∇_⊥_Ψ=−*e*(*E*_*r*_−*B*_*θ*_). It therefore follows that ∇_⊥_*F*_*z*_=∂_*ξ*_*F*_⊥_ which is the P–W theorem for both linear and nonlinear wakes. In other words, if one can measure the longitudinal variation of the focusing force, one can deduce the transverse variation of the accelerating/decelerating force or vice versa. For the special case of a fully blown-out wake that is azimuthally symmetric, Gauss' law gives 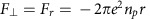 so that ∂_*ξ*_*F*_⊥_=0, whence ∂_*ξ*_*F*_*r*_=∂_*r*_*F*_*z*_=0. Here *n*_*p*_ is the ion density corresponding to the plasma density. Such a wake, where *F*_*r*_ is linear in *r* but independent of *ξ*, has the necessary field structure for accelerating an electron bunch without any emittance growth^19^. We find that, for much of the PWFA cavity that is devoid of plasma electrons, the transverse force is indeed constant longitudinally to within ±3% (r.m.s.). Moreover, comparison of experimental data and simulations has resulted in mapping of the longitudinal electric field of the unloaded wake up to 83 GV m^−1^ to a similar degree of accuracy.

## Results

### Field structure of the cavity from computer simulations

**Figure 1:**
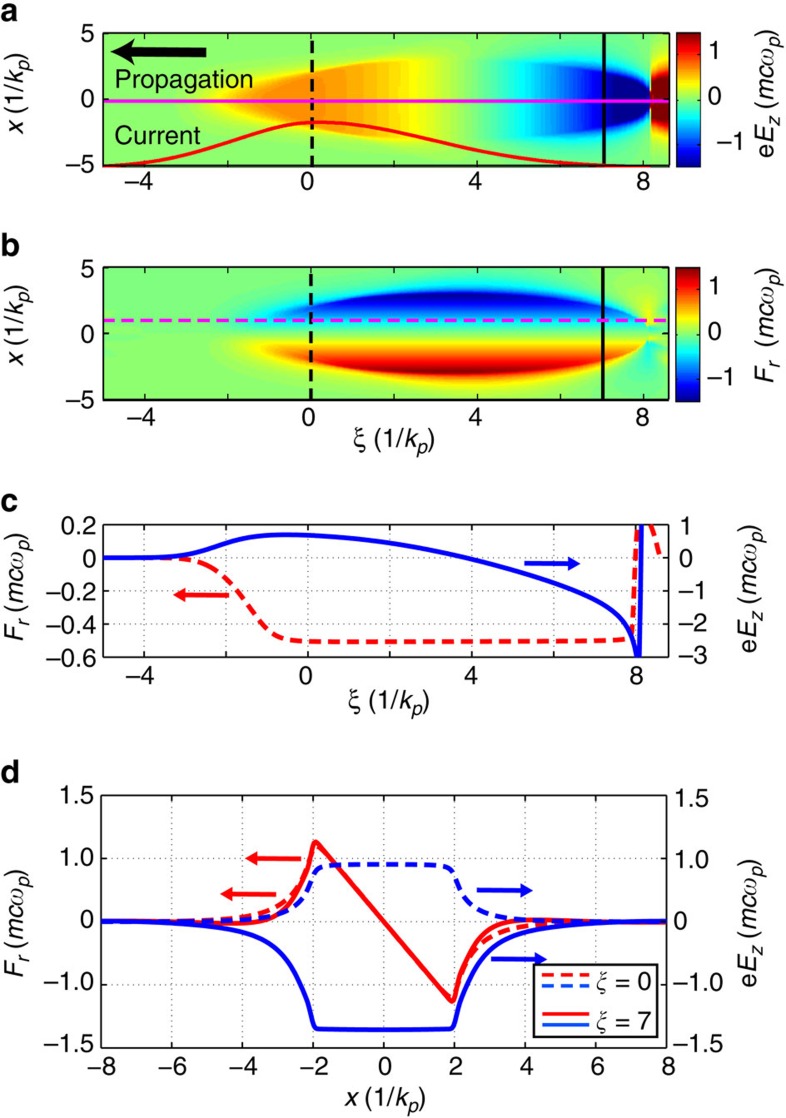
From simulation: distribution of forces within the cavity. (**a**) Longitudinal *F*_*z*_ and (**b**) transverse *F*_*r*_ force distributions of a 3D, nonlinear PWFA cavity, both shown in the *y*=0 plane and in the frame of the bunch. The black arrow in (**a**) shows the propagation direction of the cavity while the red curve in (**a**) shows the relative beam current along the bunch. (**c**) Longitudinal variation of *F*_*z*_ (solid curve) along the horizontal solid line in (**a**) and *F*_*r*_ (dashed curve) along the horizontal magenta dashed line in (**b**). (**d**) The transverse variation of *F*_*r*_ and *F*_*z*_ across the cavity at *k*_*p*_*ξ*=0 (dashed curves corresponding to the two dashed vertical lines in (**a**,**b**) and at *k*_*p*_*ξ*=7 (solid curves corresponding to the two solid vertical lines in (**a**,**b**). The normalization *mcω*_*p*_ corresponds to about 48 GeV m^−1^ for this density.

Particle-in-cell code^20^,^21^ simulations (Fig. 1a,b) show the distribution of *F*_*z*_ and *F*_*r*_ in the (*x*, *ξ*) plane at *y*=0, respectively (see Methods for simulation details). The horizontal lines indicate the *r*-locations for the lineouts of these forces that are plotted in Fig. 1c. Similarly, the vertical lines in Fig. 1a,b indicate the *ξ*-locations of the lineouts of these forces shown in Fig. 1d. It can be seen that, in the blow-out regime of the PWFA (*ξ*≳0 here) *F*_*r*_ is constant with *ξ* (dashed curve in Fig. 1c) and varies linearly with the transverse coordinate *r* within the wake as seen by the *F*_*r*_ lineouts at *k*_*p*_*ξ*=0 and 7 (red curves in Fig. 1d). To preserve the emittance of a trailing bunch, it must be placed in the region where *F*_*r*_ is linear; that is, where the motions of particles with a given energy but different transverse positions are correlated^19^. Furthermore, the blue curves in Fig. 1d, show that *F*_*z*_ is uniform in *r* in the blow-out region, changing from decelerating at *k*_*p*_*ξ*=0 to accelerating at *k*_*p*_*ξ*=7, allowing all the particles in a particular longitudinal slice to lose or gain energy at the same rate regardless of their transverse position. In this paper, we present single-shot experimental results that give quantifiable information about the longitudinal field/force distribution within the highly nonlinear wake that are consistent with predictions of the above simulations.

### Experimental overview

In the experiment, we use a dense (*n*_*b*_>*n*_*p*_), short (*k*_*p*_*σ*_*z*_<1) and ultra-relativistic (*γ*>>1) electron bunch that is tightly focused (*k*_*p*_*σ*_*r*_<1) in a plasma to excite a 3D, nonlinear wake. Here *k*_*p*,_ *σ*_*r*,_ *σ*_*z*_ and *γ* are the wavenumber, the r.m.s. spot size and bunch length, and the Lorentz factor, respectively. The single bunch (at 1 Hz) from the facility for advanced accelerator experimental tests (FACET) at the SLAC National Accelerator Laboratory^22^—having an energy of 20.35 GeV, ∼30 μm *σ*_*r*_ and ∼25 μm *σ*_*z*_ and containing 2 × 10^10^ electrons with a normalized emittance of 200 × 50 μm (*ɛ*_*x*_ × *ɛ*_*y*_)—is used to produce the plasma, drive the wake and probe the fields of the wake. The 2.5 × 10^17^ cm^−3^ density plasma is formed by field ionization of a column of Li vapour by the electric field of the electrons in the very front of the bunch^23^,^24^. The Li vapour column has a 27 cm flat-topped region with 5-cm (half-width at half maximum) up- and down-ramps. Since *n*_*b*_>*n*_*p*_, the electrons from the singly ionized Li atoms are expelled transversely by the field of beam electrons leaving a fully evacuated cavity structure that comprises plasma ions surrounded by a thin sheath of returning Li electrons. While the cavity is still forming, the bunch electrons initially experience a *ξ*-dependent focusing force and then a constant focusing force once all the plasma electrons are fully blown out which occurs for *ξ*≳0 as shown in Fig. 1c. Since the beam electrons are highly relativistic, there is negligible relative motion either between the particles themselves or between the particles and the wake; that is, the electrons see a stationary cavity. However, different slices of the bunch gain energy at different rates (see e*E*_*z*_(*ξ*) in Fig. 1c) and therefore execute a different number of spot size *σ*_*r*_ oscillations^25^,^26^ induced by the focusing force of the plasma ions. These slices undergo from 0 up to 27 spot-size oscillations depending on their energy and the strength of the focusing force. Once the cavity is fully formed so that *F*_*r*_ is constant, the accumulated phase advance Φ(*ξ*) of the oscillations depends on the final energy of the electrons of a given slice, which in turn only depends on *E*_*z*_(*ξ*) and the interaction length. If one can unambiguously identify these energy-dependent oscillations once the bunch exits the wake, one can reconstruct *E*_*z*_(*ξ*).

### Theory of slice spot-size oscillations and divergence

**Figure 2:**
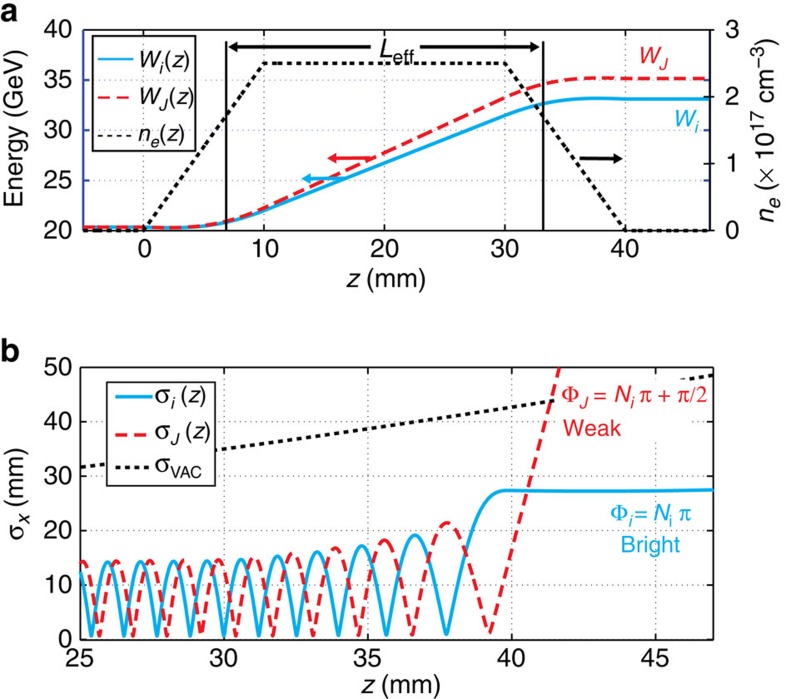
Results from numerical modeling: electron energy and spot-size variations. (**a**) Energy evolution versus *z* for slices at *ξ*_*i*_ (blue solid curve) and *ξ*_*J*_ (red dashed curve) having final energies of *W*_*i*_ and *W*_*J*_, respectively, along with the electron density profile (black dotted curve). For clarity, the evolution for slice *k* is not shown. The effective flattop length (*L*_eff_) is indicated and includes portions of the ramps. While *L*_eff_ is representative of the effective acceleration length, proper inclusion of the ramps will affect the numerical values of the divergence angles *θ*(*ξ*) of the different slices but not the energies at which *θ*_min_ occurs and therefore the conclusions drawn from this methodology still hold. (**b**) Calculated transverse spot-size *σ*_*r*_ oscillations for the two initially mismatched bunch slices (both initially 25 μm r.m.s focused at *z*=5 mm) as they propagate through the plasma at two different accelerating gradients: e*E*_*z*_(*ξ*_*i*_) (blue curve) and e*E*_*z*_(*ξ*_*J*_) (red curve). Large- (small-) divergence angles appear as Weak (Bright) charge variation on the spectrometer screen (see text). For this computation, *W*_*i*_ and *W*_*J*_ are 33.1 GeV and 35.2 GeV, respectively. The dotted black curve shows the variation of the beam size in the absence of plasma.

Consider an unmatched electron bunch that has a waist at the entrance of a slab of plasma with length *L*_eff_ as shown as in Fig. 2a. For simplicity we consider three slices (only two are shown in Fig. 2a) that reside in the region of constant *F*_*r*_ and negative (accelerating) *E*_*z*_ having subscripts (*i*, *J*, *k*) with slice *i* exiting the wake first and slice *k* exiting the wake last. The divergence angle of slice *i*, *θ*_*i*_(*ξ*_*i*_) (at a large distance from the exit of the wake), is given by d*σ*_*r*_ (*z*, *ξ*_*i*_)/d*z* and depends on the phase advance of it's oscillations given by 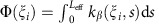 radians. Here, 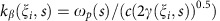 is the wavenumber of the oscillation so that^25^


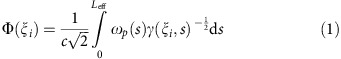


with 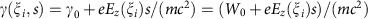, where *k*_*β*_=*ω*_*β*_/c and *ω*_*β*_ is the betatron frequency given by 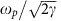, where *ω*_*p*_ is the plasma frequency and represents the square root of the ion density in the wake, *W*_0_=*γ*_0_*mc*^2^ is the initial bunch energy while *γ*(*ξ*_*i*_, *s*) is the energy of a beam slice at the coordinate (*ξ*_*i*_, *s*) where *s* is the propagation distance. If this slice exits the wake with phase advance Φ(*ξ*_*i*_)≈*πN*_*i*_, it will exit near a spot-size maximum so that *θ*_*i*_ will be extremely small as shown by the blue solid curve in Fig. 2b. The final energy of this slice is *W*_*i*_. Here, *N*_*i*_ is the total number of spot-size oscillations undergone by slice *i*. Now, the later slice at *ξ*_*J*_ will exit the wake having necessarily a higher energy *W*_*J*_ and thus will have undergone a smaller phase advance Φ(*ξ*_*J*_). If Φ(*ξ*_*J*_)=Φ(*ξ*_*i*_)−*π/2=π*(*N*_*i*_−1/2), then this slice will have a minimum spot size with an accompanying large *θ*_*J*_ (red dashed curve in Fig. 2b). These two slices are illustrated in Fig. 2b as ‘Bright' and ‘Weak' for slices *i* and *J*, respectively. It is the settings of—and the transport to—the spectrometer that results in the brighter spectral visibility of energy features with small exit angles because these features are relatively unaffected by the imaging energy setting of the spectrometer. This bright/weak behaviour continues so that when slice *k* (not shown) exits the wake with even higher energy *W*_*k*_ and a phase advance of Φ(*ξ*_*k*_)=*π*(*N*_*i*_−1)–an integer-*π* difference from Φ(*ξ*_*i*_)—it will be at a spot-size maximum and will also appear as a bright feature on the spectrometer screen.

### Interpretation of experimental data

**Figure 3:**
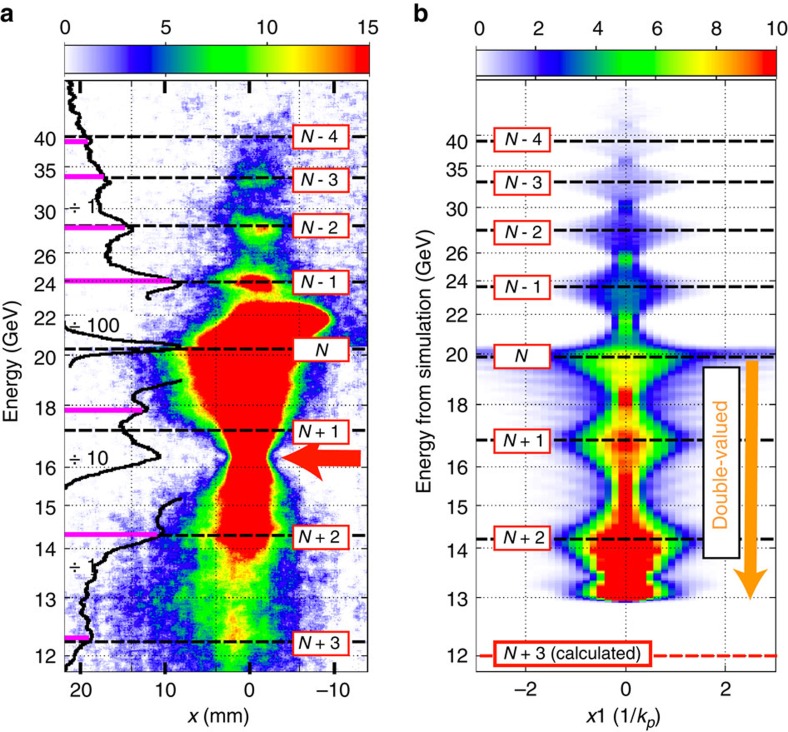
Comparison of energy spectra from experiment and simulation. (**a**) An electron spectrum (energy versus *x*) measured by the imaging spectrometer set to image electrons at 16.35 GeV (red arrow) and its *x*-integrated lineout (solid black line segments, attenuated by factors of 1, 10 and 100 as indicated). The colour table (counts per CCD pixel) is set for the energy-gain portion of the spectrum (see Supplementary Fig. 3 for a colour table set for the portion below 20.35 GeV). Also shown are the experimentally identified energy features (magenta bars) taken as the peaks in the *x*-integrated lineout (after subtracting the slowly varying background) as well as the locations of expected energy features (dashed black lines labelled *N*−4 through *N*+3, obtained through equation (2) (see text). (**b**) Energy-dependent modulations of the transverse size at the plasma exit observed in the QuickPIC simulation of the experiment with a colour table indicating the number of simulation particles per bin. The linear energy scale from QuickPIC has been mapped to a spatial scale using the experimentally determined dispersion of the spectrometer that produced (**a**). Note that the energy-loss portion of the spectrum is double valued meaning that the charge at each value of energy comes from two *ξ* locations with the wake (see Fig. 4 for the shape of the decelerating field). The energy locations of the (locally) largest transverse size of slices (that is, minimum slice divergence) upon leaving the plasma are also indicated (dashed black lines labelled *N*−4 through *N*+2, obtained by analysis of this phase-space data) and are very closely matched to the experimentally identified energy features in (**a**). Equation (2) was used along with these energy locations to predict the *N*+3 location at 12.0 GeV as shown as shown by the red dashed line. See Supplementary Fig. 4 for a ‘simulated spectrum' using the data of (**b**).

Fig. 3a shows the measured energy spectrum of the electron bunch after propagating through the Li plasma (see Methods for acquisition of electron spectra). We note that there are four bright features, labelled *N*-1 through *N*-4, in the portion of the spectrum where the electrons have gained energy. As described above, we assume that the bright features above *W*_0_=20.35 GeV are separated by a phase advance of *π* radians. We can use the relative energies of these features and equation (1) to find the expected energies of all the longitudinal slices that execute an integer number of oscillations *N*_*m*_. We calculate these energies as follows. Integrating equation (1) for an arbitrary slice within the full blow-out region gives


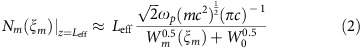


where 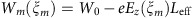 is the final energy at *z*=*L*_eff_. In this integration, we have assumed a non-evolving wake and that the ion density is constant (*F*_*r*_∝*r*) for the spectral features above *W*_0_.

To obtain an estimate of *L*_eff_, we may take any two accelerated charge features in Fig. 3a (which are separated by integer-*π* radians of phase) and use equation (2) to eliminate *N*_*m*_ (see Supplementary Fig. 1 for additional samples of measured electron spectra of which that in Fig. 3a is merely representative). For example, taking the *N*-3 and *N*-1 features at 33.6 GeV and 24.0 GeV, respectively, having a phase difference of 2*π* radians, gives *L*_eff_=22.5 cm. Note that *L*_eff_ is shorter than the 27 cm long flat-topped region of the Li vapour. The beam forms the plasma and the cavity, and acceleration continues until the phenomenon of head erosion^27^ halts the acceleration and limits the acceleration length to *L*_eff_ (which takes into account the integrated phase advance that the bunch slices experience in traversing the up-ramp), though for slices that gain energy, this is dominated by flat density region^28^. Other combinations of the four bright features above *W*_0_ give nearly the same *L*_eff_ to within 2% (mean deviation (MD)) (see Methods on statistical terminology) meaning that all four features above *W*_0_ have have been accelerated over the same distance. We then use this value of *L*_eff_ in equation (2) to predict the energies of all the beam slices that undergo an integer number of oscillations as shown by the dashed black lines labelled *N*-4 to *N*+3, overlaying the electron spectrum in Fig. 3a. In addition to the four distinct features above *W*_0_, there are three more identifiable features seen in the black lineouts (plotted to the left of the spectrum) of the spectrum near 17.8, 14.3, and 12.3 GeV (see Supplementary Fig. 3 for an unsatureated veiw of this energy-loss portion of the spectrum). One can see that the observed positions of the spectral peaks, indicated by the horizontal magenta bars and also plotted to the left of the spectrum, match extremely well with the predicted energy positions to within 1% (MD) for the seven features identified in Fig. 3a.

**Figure 4:**
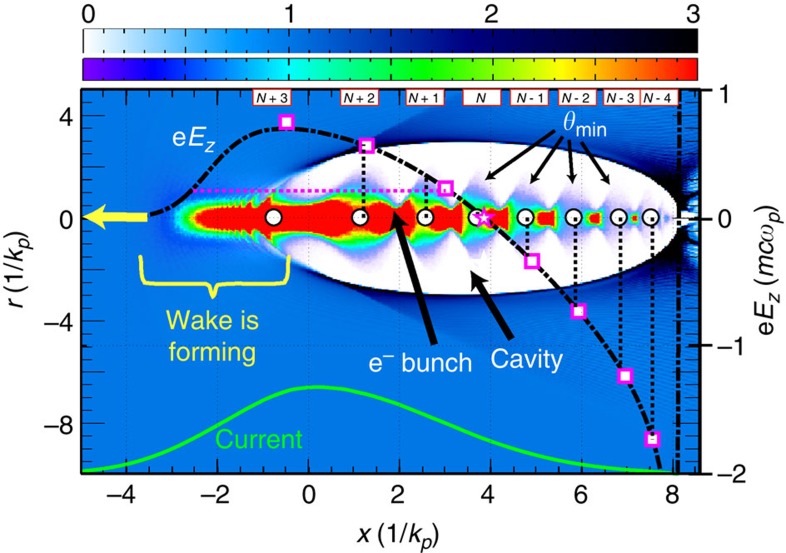
Reconstruction of the longitudinal variation of *F*_*z*_=−e*E*_*z*_. Computer simulation showing the modulation of the beam density (see the violate-to-red colorbar in units of beam density over original plasma density; the image is saturated where the current is large to bring out the tail of the beam) indicated by the arrow ‘e^−^ bunch' and the plasma wake structure (see the white-to-black colorbar in units of plasma density to original plasma density) at the end of 22.5 cm of beam propagation. The structure propagates to the left as indicated by the yellow arrow. Superimposed on this are the normalized e*E*_*z*_(*ξ*, *y*=0) (dash-dotted curve, using the scale at the right side) and the relative bunch current distribution (solid curve at bottom) as a function of *ξ* generated using the beam and plasma parameters stated in the text. The open circles indicate the positions of the beam slices at the exit the plasma having a (local) minimum *θ*(*z*=*L*_eff_). Several of these are indicated by the arrows labelled *θ*_min_(*ξ*). The heights (and thus the unique *ξ*-positions) of the vertical dotted lines correspond to the calculated, *z*-averaged e*E*_*z*_ taken from the identified energies in Fig. 3b divided by *L*_eff_ and are labelled *N*+3 through *N*−4. The magenta squares (and the star at *N*) indicate the experimentally identified positions of the energy peaks, as shown in Fig. 3a, after conversion into e*E*_*z*_ (see text). The horizontal dotted magenta line indicates that the *N*+1 feature can be affected by electrons near the rising edge of bunch. The range of *ξ* where the wake is forming is indicated. The arrow labelled ‘cavity' indicates the region where the plasma electrons have been fully blown out. All e*E*_*z*_ quantities have been normalized to *mcω*_*p*_≈48 GeV m^−1^ at *n*_*p*_=2.5 × 10^17^ cm^−3^.

The particles close to 20 GeV are comprised of a portion at the very front of the bunch expanding at near the vacuum expansion rate (where *F*_*r*_∼0) and slices near the zero crossing of *F*_*z*_ (thus staying near 20 GeV) and forming the feature labelled *N* having 24 oscillations. This feature, marked by a star in Fig. 4, separates the range in *ξ* for energy gain and loss and is located at *E*_*z*_=0. The *N*+1 peak at 17.8 GeV, shown in Fig. 3a, has the largest difference (4%) from the expected energy location. This is possibly due to it being affected by many closely spaced peaks near the front of the bunch (having nearly the same energy) when the plasma electrons are still being blown out and the ion cavity is still forming (see dotted, horizontal magenta line in Fig. 4).

In this single-shot spectrum, *L*_eff_ is fixed and thus the observed variation of the energy locations of the identified features with respect to the expected energy locations is in fact due to a variation of *F*_*r*_ via a variation of *ω*_*p*_^2^; that is, the degree of blow-out along *ξ*. For the *N-*1 to *N*-3 features used here *F*_*r*_ is essentially 1, where unity corresponds to the force due to a pure ion column of density 2.5 × 10^17^ cm^−3^. The spread in *F*_*r*_ is due to experimental limitations (see Supplementary Fig. 2 for *F*_*r*_ distribution and Supplementary Discussion for errors in calculating the restoring force *F*_*r*_). Although this analysis is for a single shot, for the data set as a whole, analysis of 33 combinations of such spectral peaks above *W*_0_ gives *F*_*r*_=1±3.0% (r.m.s.). In other words, in the accelerating portion of the wake, ∂_*ξ*_*F*_*r*_≈0. Since, ∂_*ξ*_*F*_*r*_=∂_*r*_*F*_*z*_ from the P–W theorem, we conclude that ∂_*r*_*F*_*z*_=0 must also be true to a similar degree of uncertainty.

### Mapping the longitudinal field structure

In Fig. 3b we show the energy spectrum obtained using the computer code QuickPIC using the same plasma and beam parameters as in the experiment except we assumed a plasma with a flat density profile (of length *L*_eff_=22.5 cm) as in the above model and a bi-Gaussian electron bunch with a 20 μm r.m.s. rise and a 30 μm r.m.s. fall. This energy spectrum shows energy-dependent spot-size modulations at nearly identical energies as observed in the experimental spectrum of Fig. 3a. Note that the energies of these features are insensitive to the precise transverse parameters in the QuickPIC model as long as the accelerating cavity reaches blow-out by approximately the peak of the current profile. The excellent agreement (except for the *N*+3 peak) between the experiment, the theoretical model and the self-consistent simulations proves that the modulations are caused by the longitudinally constant focusing force—∂_*ξ*_*F*_*r*_=0—of the ions in the accelerating cavity. The *N*+3 peak, only 1 GeV below the *N*+2 peak, is missing in the simulations because small changes to the rise time of the bunch in the simulation leads to changes of this scale in the maximum energy loss of the particles without significantly affecting the energy gain. Taken together Fig. 3a,b shows an excellent agreement between the experiment, theory and simulations in predicting the energy-dependent spot-size oscillation peaks.

The dash-dotted curve in Fig. 4 shows the normalized accelerating field overlaid on the beam density and the plasma cavity at the end of the simulation; that is, at *z*=*L*_eff_. The energies of the spot-size modulations from Fig. 3b were mapped to their local field *E*_*z,*sim_(*ξ*) by dividing those energies by *L*_eff_ and as such these are averaged over *L*_eff_. These *E*_*z,*sim_(*ξ*) are indicated by the vertical dotted black lines in Fig. 4. The open black circles, plotted on top of the beam density, indicate the local spot size maxima of the beam and thus its local minimum exit angles, *θ*_min,sim_(*ξ*), at the instant the simulation reached *z*=*L*_eff_. The fact that the averaged *E*_*z,*sim_(*ξ*) match up with the instantaneous *θ*_min,sim_(*ξ*) shows that the wake does not evolve significantly over the simulation length. Finally, using the analogous method for the experimental data, we take all the experimental energy features from Fig. 3a and convert them into a normalized field gradient *E*_*z,*exp_=e*E*_*z,*exp_/*mcω*_*p*_ by dividing their energy values by *L*_eff_ . For *E*_*z*_ beyond its maximum value, *E*_*z*_ monotonically decreases and therefore there are unique *ξ*-intercepts of the experimental values of *E*_*z,*exp_ and *ξ*. These are indicated by the magenta squares. The fact that the vertical lines, derived from simulation, intercept these magenta squares from experimental data to within 1.9% (1.4% MD) shows that longitudinal field structure of the wake has been accurately mapped using this technique. Note that the peak decelerating field is 36 GV m^−1^ while the accelerating field at the *N*-4 peak is −83 GV m^−1^. With such detailed information of the actual shape of the field, one can place an optimized shaped trailing bunch to load or flatten the wake for preserving the energy spread of the bunch and efficiently extract energy from the wake^12^.

## Discussion

To conclude, we have mapped the longitudinal variation of the *F*_*z*_ and *F*_*r*_ forces of a fully blown out PWFA using the drive electron bunch itself to probe the field structure of the cavity. We have shown that for such a cavity (*ξ*≳0 here), ∂_*ξ*_*F*_*r*_=0 within the measurement accuracy of about ±3% (r.m.s). This is because the cavity is comprised of a uniform density of plasma ions that exert a linear focusing force on all the beam slices that are in the accelerating phase and blown out region of the wake. This in turn implies that *∂*_*r*_*F*_*z*_ is also equal to zero and that all the particles in a given slice are accelerated at the same rate. This ability to map the field structure is essential for optimizing the bunch shape so as to load a matched trailing electron bunch for high-gradient, high-efficiency acceleration of a narrow energy spread beam^12^ while undergoing little emittance growth in such a PWFA cavity^19^.

## Methods

### Computer simulations

Computer simulations were carried out with the 3D quasi-static particle-in-cell code QuickPIC^20^,^21^. The simulation box tracks the beam–plasma interaction in the speed-of-light coordinates *x, y, ξ*=*z−ct*. The box has a size of 500 μm × 500 μm × 192 μm in the two transverse dimensions and the longitudinal dimension, respectively. The number of cells for the simulation box is 1024 × 1024 × 512 (∼0.54 billion cells in total). The code used the same plasma and the beam parameters as in the experiment except we assumed a plasma with a flat density profile (of density 2.5 × 10^17^ cm^−3^ and length *L*_eff_) as in the model of equation (2). To pre-compensate for the lack of quasi-adiabatic focusing in the up-ramp, the bunch with a normalized emittance of 130 × 130 μm (*ɛ*_*x*_ × *ɛ*_*y*_) containing 2 × 10^10^ electrons (8.4 × 10^6^ beam particles in the simulation) began with a round, transverse spot of *σ*_*r*_=7 μm r.m.s. The results were not sensitive to this exact choice of *σ*_*r*_. The longitudinal distribution of the single, 20.35 GeV bi-Gaussian electron bunch was 20 μm r.m.s. rise and a 30 μm r.m.s. fall. The code uses the quasi-static approximation, which assumes the beam evolves slowly compared with the transit time of a plasma electron through the region of the bunch. However, the forces on the beam were updated many times per betatron oscillation. Thus, a snapshot of the particles and fields was recorded approximately every 1 mm. The fields in Fig. 1 are taken from the simulation at a distance of *L*_eff_ of about 22.5 cm. The particle data of Fig. 3b and Fig. 4 are taken at this same position in the plasma. Note that, in Fig. 1c, *E*_*z*_ is double valued for −3≲*ξ*≲+4 and the corresponding range of energies is labelled in Fig. 3b. The ‘fishbone-like' structure below 20.35 GeV is due to the fact that the wake is still forming for −3≲*ξ*≲0 .

### Acquisition of electron spectra

The electron spectrometer has been described in detail in Adli *et al.*^29^. The physical height of the entire dispersed spectrum required the use of two 12-bit charge-coupled device (CCD) cameras; one viewing from 17 to 63.8 GeV (the upper range of energies) and the other having an overlapping range of 11.7–23.7 GeV (the lower range of energies). Each of the two cameras had a background image taken just before acquiring a sequence of data and these were subtracted from the data images. The image in Fig. 3a of the main text contains spectral data from both of these CCD cameras: 11.7 GeV to *W*_0_=20.35 GeV from the lower-range camera and *W*_0_ to 63.8 GeV from the upper-range camera albeit cropped at 50 GeV. A very small discontinuity (on the order of a few counts per CCD pixel) between the two images at *W*_0_ is visible and this is believed to be due to the two cameras having slightly different light-collection efficiencies. The very low range of CCD counts, typically less than 30 counts above background, necessitated the use of a median filter. Due to apparent damage to these cameras, randomly distributed noise was mixed in with the image of the spectrum. A histogram of a region in a corner of the image showed the expected peak in the 1–2 CCD-count bin, but with an underlying, near-gaussian distribution of noise having a r.m.s. width of 8.8 counts and thus a large percentage of pixels had as much as 20 counts of noise. A median filter was chosen to be a square of 21 pixels by 21 pixels in which each pixel value was replaced by the average of those in the 21 by 21 neighbourhood surrounding that pixel. The result is what is shown in Fig. 3a. This filtering did not significantly change the size of the energy features and, more importantly, did not change their location. The transverse size of the boxout that was integrated to produce the solid black curve in Fig. 3a covered −5 mm<*x*<5 mm.

### Statistical terminology

The ‘r.m.s.' is used often in this manuscript. However, for sets of numbers which are far from a normal distribution or contain too few points, the r.m.s. measure is inappropriate. For those number sets, it is common to specify the mean of the absolute deviation of each number from the average of the set. This ‘MD' (also referred to by ‘mean absolute deviation' (MAD)) of a data set *A* is defined as 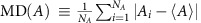, where 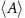 is the average of that data set containing *N*_*A*_ elements. The MD does not use a ‘±' since it is an absolute-value deviation.

### Data availability

The data that support the findings of this study are available from the corresponding author upon request.

## Additional information

**How to cite this article:** Clayton, C.E. *et al.* Self-mapping the longitudinal field structure of a nonlinear plasma accelerator cavity. *Nat. Commun.* 7:12483 doi: 10.1038/ncomms12483 (2016).

## Supplementary Material

Supplementary Information Supplementary Figures 1-4 and supplementary Discussion.
