# Supplementary material for: Self-mapping the longitudinal field structure of a nonlinear plasma accelerator cavity
Source: Nat Commun. 2016 Aug 16;7:12483. doi: 10.1038/ncomms12483 (PMC4990705; doi:10.1038/ncomms12483)
Supplement: Supplementary Information — Supplementary Figures 1-4 and supplementary Discussion. [file ncomms12483-s1.pdf]

Additional samples of measured electron spectra

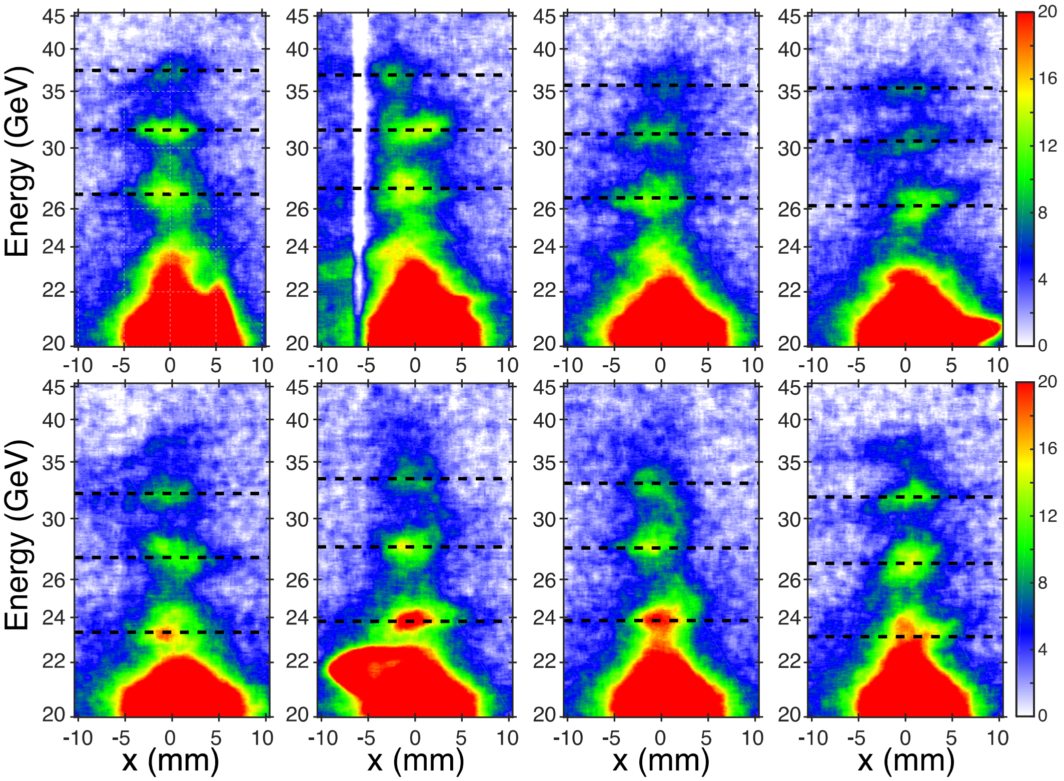

Supplementary Figure 1. Additional samples of measured electron spectra. Selection of eight events showing the identified features (dashed black lines) above the initial energy W_0 = 20.35 GeV. Upper row: events that required using the highest visible energy features (> 35.GeV) to obtain three clean measurements of the energy features (lower energies are obscured by kicked/defocused electrons up to near 22 GeV). Lower row: events where spectral features just above 22 GeV were used due to a higher signal-to-noise (S/N) here. For all shots analysed, the mean estimate of the plasma length L_{eff} varied from 22.5 cm to 25.6 cm with the upper (lower) row having the longer (shorter) effective lengths. For these shots and the event in Fig. 3a of the main manuscript, there appears to be "excess charge" from just below W_0 up to almost 22 GeV. In the experiment, the electron bunch had a low-current "nose" to it (at W_0), which is kicked by the formation of the wake causing excess charge at W_0. Since the spectrometer was focused for 16.35 GeV, this angular kicks "look like" energy change with the kicked nose mainly presenting as background near W_0.

F_r distribution

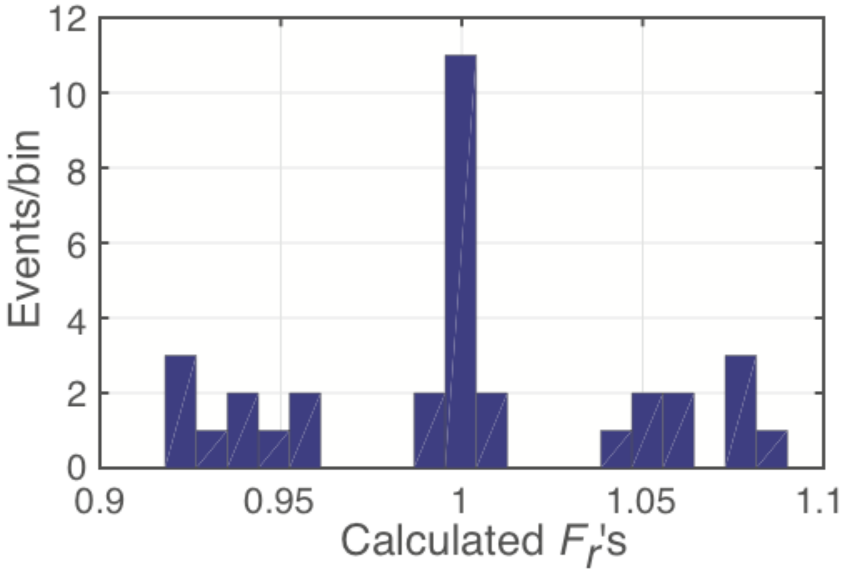

Supplementary Figure 2. F_r distribution. Histogram of the calculated, normalized restoring force F_r for 11 events, each event giving three estimates for F_r. Here, $F_r = 1.0$ corresponds to an ion density of 2.5×10^{17} cm^{-3}. There are two properties of this distribution to note. First, a large fraction of the points are cantered near $F_r = 1$. Second, there are two wings, each with 11 points. The two wings are not independent. See Supplementary Discussion on the errors in calculating the restoring force F_r.

Exprimentally-identified energy features below 20.35 GeV

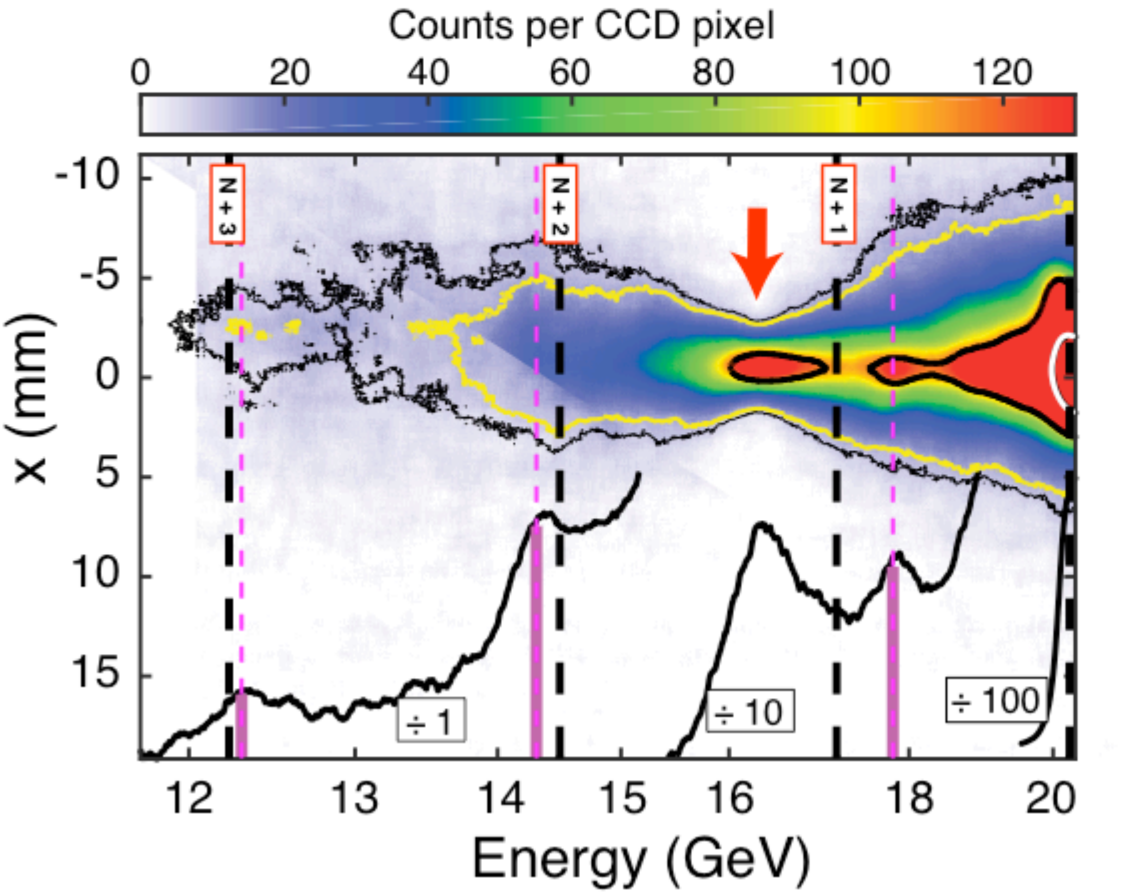

Supplementary Figure 3. Experimentally identified energy features below 20.35 GeV. The same electron spectrum (energy vs. *x*) from the imaging spectrometer as in Fig. 3a of the main manuscript measured by the imaging spectrometer set to image electrons at 16.35 GeV (red arrow) and its x-integrated lineout (solid black curve segments, attenuated by factors of 1, 10 and 100 as indicated). The color table (in counts per CCD pixel) was too low to see these features in Fig. 3a. It is now increased by a factor of ~ nine and, with the addition of contours–[thin black, yellow, thick black, white] = [9, 13, 115, 1200]–highlights the energy-loss portion of the spectrum. As in Fig. 3a of the main manuscript, the x-integrated lineout reveals the energy locations of the identified features, marked by the vertical magenta bars (continued by the thin, short-dashed magenta lines) while the vertical dashed black lines labelled N+1 through N+3 (calculated using equation 2) are the same as those of Fig. 3a.

Simulated spectum

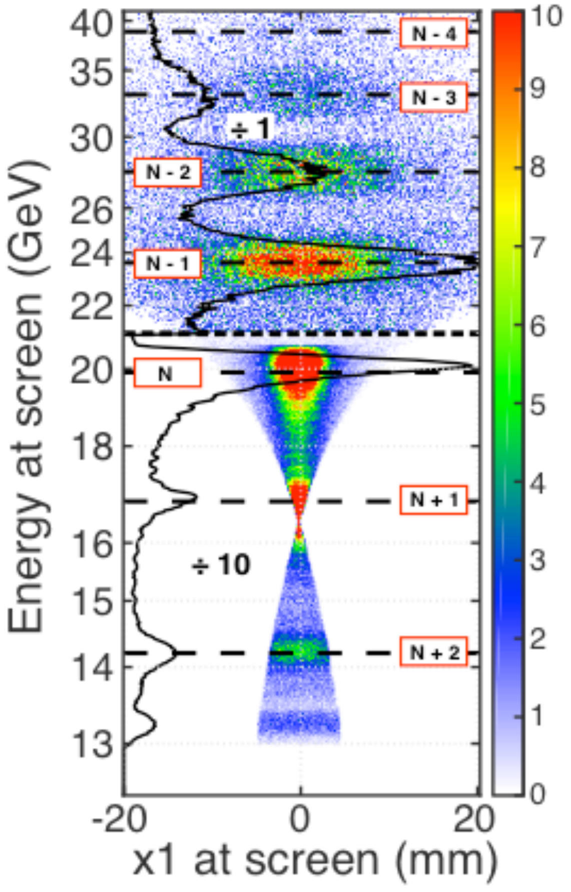

Supplementary Figure 4. Simulated spectrum. A simulated electron spectrum using the same simulation data of Fig. 3b of the main manuscript where all ~ 8.4 million particles were propagated through linear (first-order) transport matrices representing the elements of the spectrometer line comprising of two quadrapole magnets and a dipole bending magnet. Using the vertical separation of two known energy features–20.35 GeV (the largest peak) and 16.35 GeV (the location of the energy focus)–provides the dispersion needed to put an energy scale on this simulated spectrum. The transverse size of the energy focus is about 1 mm FWHM. The lower part of the spectrum is attenuated by a factor of 10 (below 21 GeV as indicated by the black dotted line) and this attenuation is also in the x-integrated lineout (black curve at the left). The black dashed lines are at the same energy locations of the (locally) largest transverse size of slices as in Fig. 3b of the main manuscript. The apparent peak near 13 GeV is just the upper portion of the N+3 feature which, as seen in Fig. 3b, is not fully formed until 12.0 GeV. The simulation did

not have a smooth density transition into vacuum. Such a transition would have allowed for the spot size of most of the slices to gradually increase and thus gradually reduce their angular spread. To mimic this angular damping, which decreases the size of the beam in the quadrapoles thus reducing chromatic aberrations, the angular acceptance of the transport line was reduced by a factor of five (although a factor of two looks similar but with more chromatic aberration). Note that the rapid spot-size modulations below W_0 = 20.25 GeV seen in Fig. 3b (at the front of the electron bunch as seen in Fig. 4) show up as intensity modulations in both the image and the lineout. Also worth noting is the fact that it was not necessary to attenuate the x-integrated lineout by 100 times, as was necessary near W_0 in Fig. 3a (see caption for Supplementary Figure 1).

Supplementary discussion

Errors in calculating the restoring force F_r.

For the event shown in Fig. 3a of the main manuscript, the deviations in energy of the *observed* energy features from those *expected* for a uniform and constant F_r = 1 restoring force (the horizontal black dashed lines) suggests that F_r is close to unity. However, more statistics are needed. A total of 33 calculations of F_r from 11 experimental spectra resulted in the histogram of Supplementary Figure 2. Note that a large fraction of the points are centered near F_r = 1 while there are two wings each with 11 events. Theses two wings are not independent. Each F_r is a solution of equation (2) of the main manuscript where w_p^2, the ion density responsible for the transverse restoring force, is calculated once we know L_{eff} and N_m. This value of w_p^2 is normalized to that for an ion density corresponding to the experimental density of 2.5 x 10^{17} cm^{-3} and thus should be unity for full evacuation of the plasma electrons. When three energy features are selected above W_0, as was done for Supplementary Figure 2, there are found three values of L_{eff} and thus three values of F_r for each data shot. One of these values is close to the mean value and the other two are a little higher and lower than the mean, thus yielding a central F_r that is close to unity and two values straddling this. If there are errors of a few pixels in choosing the peak of each energy feature, then errors in F_r accumulate. The error in identifying the energies of the features in Supplementary Figure 1 (eight of 11 are shown) is dominated by the signal-to-noise (S/N) ratio of the measurement. For the highest energy features, the S/N is dominated by the signal falling into the background of the

CCD camera counts (upper row in Supplementary Figure 1) while for the lowest energy features (above W_0), the S/N is dominated by the fact that some of these features sit atop a high signal from defocused particles near W_0 (lower row in Supplementary Figure 1). As a result, there can be errors in the exact pixel location of a chosen feature. This is further exacerbated due to the low dispersion at the higher energies. For example, a six-pixel error in choosing the location of, say, a peak near 30 GeV can change its energy by 0.2 GeV. In one case, this results in changing the mean deviation of the resultant three values of F_r about unity from 11% down to 5.4%. If errors in pixel selection were the only contribution, then the histogram of Supplementary Figure 2 should have a central distribution rather than the two wings. If, in addition to errors from the finite S/N, there were an error in the measured dispersion, then such a ~ 6 pixel error would occur for all analyzed events and lead to a three-peak distribution more similar to the histogram in Supplementary Figure 2. Although the pixel-to-energy mapping is our best estimate, if the dispersion is changed by < 1% percent, essentially all the estimates of F_r in Supplementary Figure 2 would collapse into a single peak near unity rather than a peak with two wings. Since the satellite peaks are spread out, it is concluded that the errors in calculating the restoring force F_r come from both a low S/N and from an apparent error in knowing the exact dispersion. As stated above, taking three combinations of energies from each event automatically yields a central F_r and two satellites. Since the satellites are not independent, the best we can do to specify a spread in the calculated forces is to take the 22 calculations between 0.9 and 1.0 in Supplementary Figure 2 yielding F_r = 0.97 having a mean deviation of 2.9% and a standard deviation of 3.2%.
